# Data-driven design and screening of novel *Klebsiella pneumoniae* carbapenemase-2 β-lactamase inhibitors using a generative CLM

**DOI:** 10.1039/d6ra02379g

**Published:** 2026-05-26

**Authors:** Syeda Sumayya Tariq, Muhammad Salman Haleem, Reaz Uddin

**Affiliations:** a Dr. Panjwani Center for Molecular Medicine and Drug Research, International Center for Chemical and Biological Sciences, University of Karachi Karachi-75270 Pakistan mriazuddin@iccs.edu; b School of Electronic Engineering and Computer Science, Queen Mary University of London E1 4NS UK; c Western Caspian University Baku Azerbaijan

## Abstract

The rapid emergence of carbapenem-resistant Enterobacterales, particularly among ESKAPE pathogens such as *Klebsiella pneumoniae*, has significantly compromised the effectiveness of existing antibiotics. This resistance, usually mediated by KPC-2 β-lactamase, poses a critical threat to effective antimicrobial therapy, necessitating the urgent need for novel inhibitors. In this study, a chemical language model (CLM) was developed to generate novel drug candidates against KPC-2 by integrating deep generative modeling with a SELFIES-based recurrent neural network. The CLM was trained on approximately 2.3 million ChEMBL compounds, achieving stable convergence and syntactic validity during generation. The generated molecules were then evaluated using RDKit and *in silico* ADME profiling, while Fréchet ChemNet Distance (FCD) was used to assess alignment with known drug-like chemical space. With an FCD score of 0.93, the generated compounds were found to be 100% RDKit-valid, with 71% compounds satisfying Lipinski's criteria, while only 3% were flagged as PAINS. The generated compounds were shortlisted based on multiple drug-like filters and were then docked into the KPC-2 active-site, while their binding stability and interaction profiles were further studied *via* extensive all-atom molecular dynamics simulations. Stability metrics, including RMSD, RMSF, *R*_g_, PCA and FEL were benchmarked against the clinically approved inhibitor of KPC-2, relebactam. As a result, compounds 46, 72, 75 and 88 demonstrated stable binding modes and favorable interaction profiles with key active-site residues of KPC-2. These findings establish a robust and scalable computational framework for the discovery of novel KPC-2 inhibitors, demonstrate the potential of CLMs as powerful tools for accelerating antibiotic discovery in the fight against antimicrobial resistance, and provide a generalizable strategy for targeting other critical resistance determinants. The CLM used in this study is publicly available at https://github.com/sumayya-tariq/Chemical-Language-Model-CLM-.

## Abstract 2

Using generative chemical language modeling (CLM) for the design and discovery of novel KPC-2 Inhibitors targeting *Klebsiella pneumonia*.

## Graphical abstract

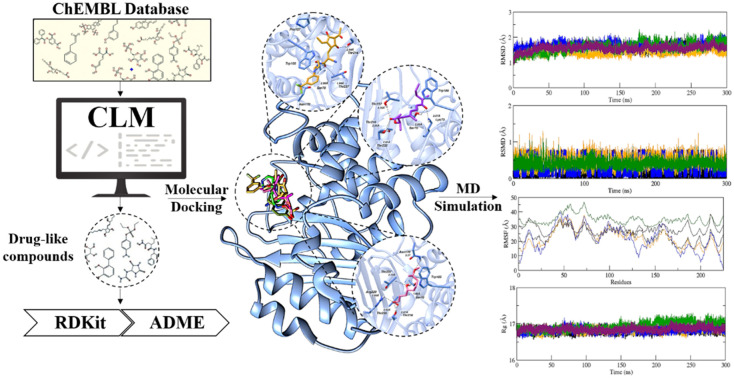

## 1. Introduction

The rapid emergence of antimicrobial resistance (AMR) represents one of the most pressing challenges to modern medicine, significantly compromising the effectiveness of existing antibiotics against life-threatening infections.^1,2^ Carbapenems are considered last-resort antibiotics for treating infections caused by multidrug-resistant Gram-negative bacteria, however, their clinical utility has been made increasingly ineffective due to the global dissemination of carbapenem-hydrolyzing enzymes. In particular, Class A carbapenemases, especially *Klebsiella pneumoniae* carbapenemase-2 (KPC-2), have been identified as major contributors to carbapenem resistance worldwide.^3,4^ Many current frontline treatments have been rendered ineffective due to KPC-2's wide substrate spectrum and ability to hydrolyze almost all β-lactam antibiotics, including carbapenems. Despite the development and approval of β-lactamase inhibitors like relebactam for clinical use, new chemical scaffolds targeting KPC-2 are required due to the persistent emergence of resistant variants and limited inhibitor coverage.^5,6^

Traditional drug discovery approaches often require extensive resources, are time consuming, and are constrained by limited opportunities of chemical space exploration, often resulting in high attrition rates during development stages.^7^ These challenges call for an increased interest in data-driven machine learning approaches to maximize lead identification and speed up hit discovery. In this regard, AI based generative models grounded in deep learning are turning out to be promising tools for *de novo* molecular design.^8^ CLMs (Chemical Language Models) represent a class of generative models that treat molecular structures as sequential data and learn the conditional probability distribution of molecular tokens, enabling the generation of novel compounds with desired properties.^9^ While early CLMs relied predominantly on SMILES representations of chemical compounds, issues related to syntactic invalidity, leading to unsound molecule generation have limited their robustness.^10^ To address these challenges, SELFIES (Self-Referencing Embedded Strings) are being used as a chemically constrained molecular representation that guarantees syntactic validity upon decoding, making them particularly appropriate for *de novo* molecular generative models.^11^ SELFIES-based CLMs have been reported to improve molecular validity and sampling stability while allowing the exploration of diverse chemical space.^12^ An example of such a models is DRAGONFLY, which integrates graph neural network-encoded receptor information with an LSTM trained on SELFIES distributions, and Conditional Variational Autoencoders (CVAE-SELFIES) employed for multi-target *de novo* drug design.^13,14^ Transformer-based CLMs such as GP-MoLFormer,^15^ GMTransformer,^16^ and cMolGPT,^17^ offer strong generative performance but also require extensive computational resources and complex training pipelines, in contrast, LSTM-based architectures operating on SELFIES provide a computationally efficient alternative without sacrificing chemical validity. The present work employs an LSTM-based CLM trained on SELFIES representations of domain-specific antibacterial compounds, combining molecular validity, computational tractability, and targeted chemical space exploration, a combination not previously applied to the design of novel KPC-2 inhibitors.

For preliminary drug discovery, a preferable CLM should be lightweight, reproducible, capable of generating chemically valid and diverse molecules, while also allowing rapid exploration of chemical space without depending on complex training pipelines and extensive computational resources. To address this need, in this study, we have developed an LSTM-based CLM using SELFIES representations, combining architectural simplicity, with robustness and high chemical validity, offering an efficient framework for *de novo* molecular generation in antibiotic discovery, especially against AMR. However, generating syntactically valid molecules alone is not sufficient for practical drug discovery. Candidate prioritization requires effective assessment of physicochemical and drug-likeness properties, distributional similarity to known bioactive compounds, and structure-based evaluation against the intended biological target.^18^

The proposed CLM is trained on ChEMBL, a large dataset of bioactive molecules with drug-like properties, to generate novel, and synthetically accessible drug-like molecules against KPC-2 β-lactamase. The generated compounds were systematically evaluated *via* RDKit for drug-likeness, Fréchet ChemNet Distance (FCD) analysis to assess distributional similarity to known chemical space, and *in silico* ADME profiling to prioritize compounds with favorable pharmacokinetic characteristics against the KPC-2 active site. The short-listed candidates were further subjected to molecular docking and molecular dynamics simulations to evaluate the stability and persistence of protein–ligand interactions. By integrating generative modeling with multiple filtering criteria, structure-based screening, and molecular dynamics simulations, this study presents a robust and scalable computational approach for identifying novel KPC-2 inhibitors, highlighting the importance of CLMs as effective tools to accelerate antibiotic discovery against antimicrobial resistance, and offer a broadly applicable strategy for addressing other resistant targets.

## 2. Methodology

### 2.1. Dataset preparation

A total of 2.3 million compounds were downloaded as canonicalized SMILES from ChEMBL database. Invalid entries, salts, disconnected fragments, and stereochemically inconsistent molecules were removed during the preprocessing.^19,20^ To improve robustness, all valid SMILES were converted into SELFIES (Self-Referencing Embedded Strings) representations using the official encoder. SELFIES provide a semantically constrained molecular representation that guarantees syntactic validity upon decoding, addressing a key limitation of SMILES based generative models.^21,22^ SMILES strings that failed conversion were discarded, ensuring a chemically meaningful training set. A custom vocabulary was constructed directly from the SELFIES dataset by tokenizing each string into its atomic SELFIES symbols using the official tokenizer. Three control tokens, a beginning-of-sequence token [BOS], an end-of-sequence token [EOS], and a padding token [PAD], were added to the dataset, following standard autoregressive language modeling practice.^23^ The final vocabulary consisted of these control tokens combined with all unique SELFIES tokens observed in the dataset, with consistent token-to-index and index-to-token mappings used during both training and generation. Each SELFIES sequence was encoded as a fixed-length token sequence by prepending [BOS] and appending [EOS], followed by padding to a maximum length of 128 tokens using [PAD]. Padding tokens were excluded from loss computation to prevent bias due to sequence length normalization, a common practice in neural sequence modeling, enabling efficient mini-batch training and autoregressive next token prediction.

### 2.2. Model architecture and autoregressive molecular generation

A CLM (Chemical Language Model) was developed for this study to provide a lightweight, transparent, and reproducible framework for large-scale molecular generation using SELFIES representations. The CLM is implemented as an autoregressive recurrent neural network based on a Long Short-Term Memory (LSTM) architecture,^24^ which is a well-established baseline for molecular sequence modeling. It models molecular sequences as tokenized strings and learns the conditional probability of each token given preceding context, enabling the generation of chemically valid molecules.

**Fig. 1:**
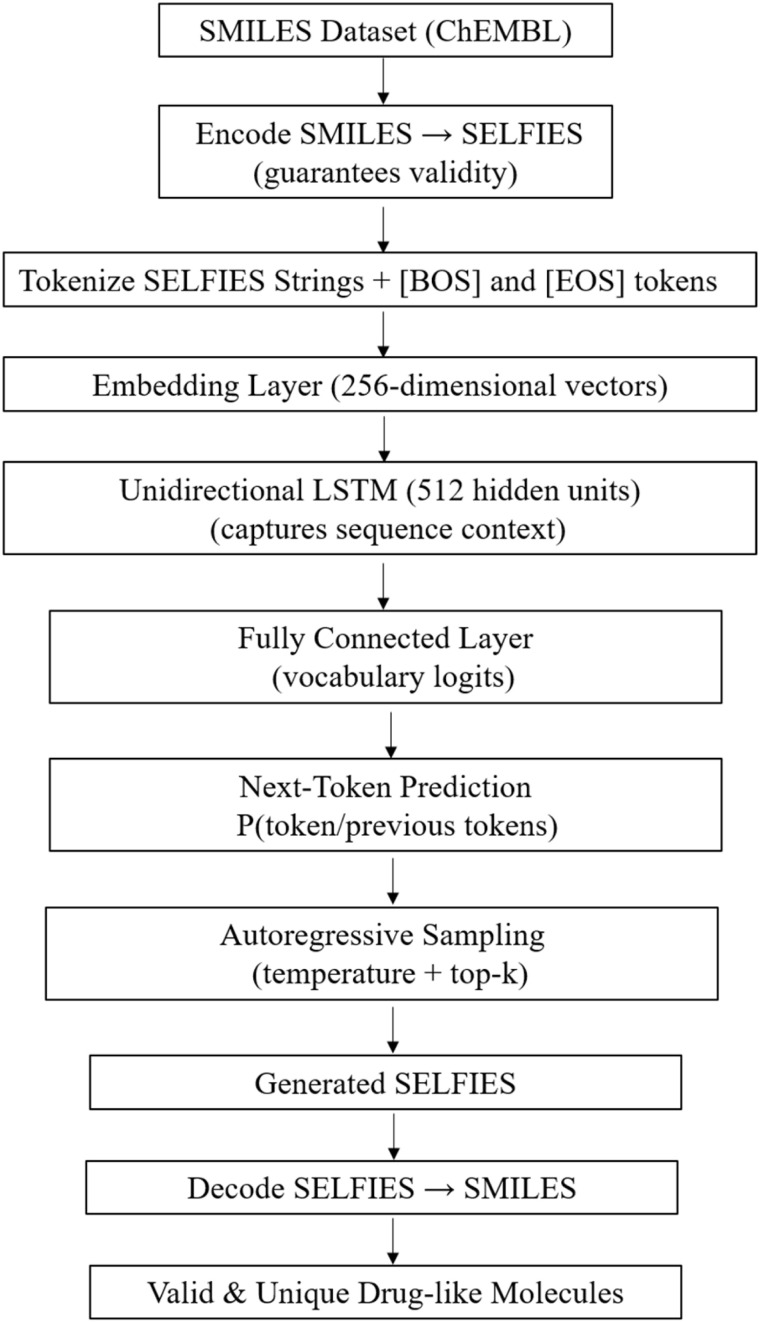
A simplified architecture of the CLM (Chemical Language Model) used in this study. Molecular SMILES are encoded as SELFIES and tokenized, then passed through an embedding layer and a single-layer unidirectional LSTM. The fully connected output predicts the next token, enabling autoregressive generation of chemically valid and diverse molecules.

The architecture consists of an embedding layer projecting input tokens into a 256-dimensional latent space, a single-layer unidirectional LSTM with 512 hidden units to capture long-range dependencies such as ring closures and recurrent functional group patterns, and a fully connected output layer mapping LSTM hidden states to vocabulary logits. This design emphasizes architectural simplicity, explicit control over each component, minimal external dependencies, and stable training on modest hardware. SELFIES representations ensure syntactic validity without post-generation correction. A simplified architecture of this CLM is presented in Fig. 1.

Mathematically, if a SELFIES string is represented as a token sequence *X* = (*x*_1_, *x*_2_, …, *x*_*T*_), the model estimates the joint probability using the chain rule:
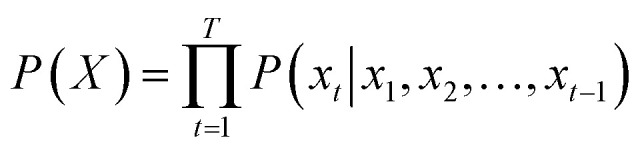


At each step *t*, the LSTM updates its hidden state *x*_*t*_, based on the current token embedding and previous hidden state, with gating mechanisms (input, forget, output, and cell state) enabling the modeling of long-range dependencies.

### 2.3. Training procedure and optimization

The dataset was randomly split into training (90%) and validation (10%) subsets. Training employed teacher forcing, optimizing the model to predict the next token given the ground-truth sequence. The Adam optimizer (learning rate 3 × 10^−4^) minimized categorical cross-entropy loss, with padding tokens masked to prevent spurious gradients. Training was conducted for 10 epochs, with token-level loss and perplexity monitored for convergence, and validation loss evaluated after each epoch to detect overfitting.^25^ Random seeds were fixed for reproducibility across Python and PyTorch backends. Training was conducted on an NVIDIA GeForce GTX 1070 GPU, though CPU execution is possible with slower performance.

For a vocabulary of size *V*, the categorical cross-entropy loss at a single time step is:
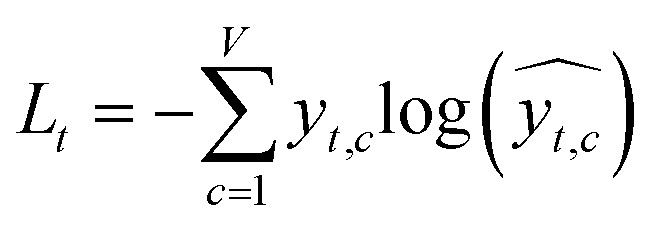
where *y*_*t*,*c*_ is the binary indicator (0 or 1) of the correct token, and 
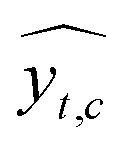
 is the predicted probability.

### 2.4. Molecule generation with top-k sampling

After training, molecules were generated autoregressively starting from the [BOS] token. At each time step, the model outputs a probability distribution over the vocabulary. Top-k sampling (*k* = 50) with temperature 1.0 was applied to retain only the most probable tokens, balancing diversity and validity. Generation terminated upon emission of [EOS] or reaching the maximum sequence length.^26^

The probability distribution over the vocabulary is computed as,
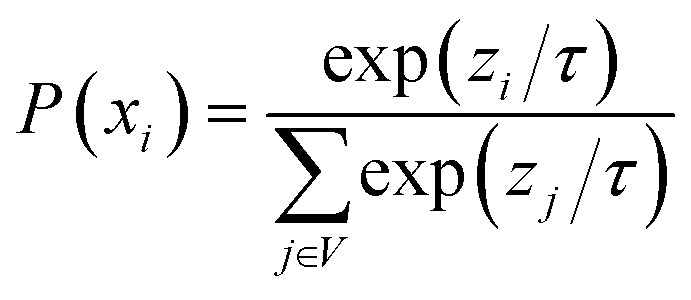
where *z*_*i*_ is the logit for token *i* and *τ* = 1.0. Top-k sampling truncates the vocabulary to the 50 most probable tokens, ensuring syntactic validity and reducing the chance of invalid molecular structures while preserving chemical diversity.

### 2.5. Distributional similarity evaluation

Fréchet ChemNet Distance (FCD) was used to evaluate how well generated molecules reproduce the statistical properties of drug-like chemical space in ChEMBL. FCD is the chemical equivalent of the Fréchet Inception Distance (FID) used in image generation.

Rather than comparing molecules atom-by-atom, FCD compares the distributions of high-dimensional features extracted from a pre-trained ChemNet network. Assuming the feature activations of the generated (G) and reference (R) datasets follow Gaussian distributions, FCD is computed from their means (*µ*) and covariances (*Σ*) as:FCD = |*µ*_g_ − *µ*_r_|_2_^2^ + Tr(*Σ*_g_ + *Σ*_r_ − 2(*Σ*_g_*Σ*_r_)^1/2^)where, |*µ*_g_ − *µ*_r_|_2_^2^ is the squared Euclidean distance between the feature means. This penalizes the model if the *average* generated molecule fundamentally differs from the average reference molecule. Tr denotes the trace (the sum of the diagonal elements) of the resulting matrices, and the covariance (*Σ*) measure the diversity of the generated molecules. If the model suffers from mode collapse (generating the same few molecules repeatedly), *Σ*_g_ will be artificially small compared to *Σ*_r_, which steeply increases the FCD penalty.^27^

### 2.6. Post-generation validation *via* RDKit

To assess the chemical validity, drug-likeness, and synthesizability of the molecules generated *via* the CLM, the generated SMILES were first parsed into RDKit. It is an open-source toolkit widely used for the analysis of chemical structures, providing functionalities for reading, descriptor generation, and processing molecular representations such as SMILES.^28^ Molecules failing parsing were classified as chemically invalid and excluded from downstream analysis, ensuring only interpretable molecular representations were considered.^29^ For each valid molecule, a set of physicochemical descriptors commonly used to characterize drug-likeness was computed, including molecular weight (MW), octanol–water partition coefficient (log *P*), number of hydrogen bond donors (HBD) and acceptors (HBA), rotatable bonds (RB), and topological polar surface area (TPSA). Lipinski's Rule of Five and Veber descriptors were applied to classify molecules as drug-like or non-drug-like, while the Quantitative Estimate of Drug-likeness (QED) provided a measure of overall drug-like quality.^30–32^ Molecules were further screened for PAINS substructures to flag potential assay-interfering compounds, and synthetic accessibility (SA) scores were calculated using a fragment-based heuristic approach to estimate ease of synthesis.^33,34^ All computed descriptors and evaluation results were exported as a structured report, facilitating systematic assessment in line with established *de novo* molecular generation benchmarks.

### 2.7. ADME evaluation and toxicity profiling

To further evaluate the pharmacokinetic suitability of the shortlisted compounds generated by the CLM, *in silico* ADME profiling was performed using the SwissADME web server (https://www.swissadme.ch) to complement the physicochemical descriptors computed locally. For this purpose, canonical SMILES of the shortlisted molecules were submitted to the server to predict key Absorption, Distribution, Metabolism, and Excretion (ADME) properties, including physicochemical attributes, pharmacokinetic parameters, bioavailability considerations, permeability across the blood–brain barrier, adherence to Lipinski's Rule of 5, synthetic accessibility, and the potential toxicity of small molecules. The outputs from SwissADME were used to rank compounds based on their predicted pharmacokinetic profiles and low ADME-related liabilities, further prioritizing them for structure based analysis. As an additional measure for confidence, toxicity profiling of the short-listed compounds was also performed using ProTox-III.

### 2.8. Molecular docking

Molecular docking was performed to evaluate the binding potential of shortlisted molecules generated by the CLM. A total of 100 compounds, shortlisted based on multiple drug-likeness filtering criteria, were subjected to docking analysis using AutoDock Vina. The selected molecules were prepared for docking by converting SMILES into three dimensional structures using RDKit. Explicit hydrogens were added, and ligand geometries were energy-minimized using the MMFF94 force field to eliminate unfavorable conformations. The optimized ligands were subsequently converted into PDBQT format, with Gasteiger partial charges assigned and rotatable bonds defined. The crystal structure of KPC-2 β-lactamase (PDB ID: 6QW9), co-crystallized with the inhibitor relebactam, was selected as the docking receptor. Prior to docking, solvent molecules and non-essential heteroatoms were removed. Polar hydrogens were added, and Kollman partial charges were assigned using AutoDock Tools, and the prepared receptor was saved in PDBQT format. A three-dimensional grid box was defined to encompass the active site of the enzyme. The grid center was positioned based on the coordinates of the co-crystallized ligand, and the grid dimensions were selected to allow adequate conformational sampling of the binding pocket.

### 2.9. All-atom molecular dynamic simulation protocol

All-atom Molecular Dynamics (MD) simulations were performed to evaluate the stability and dynamic behavior of the shortlisted KPC-2–ligand complexes, while KPC-2–relebactam complex was used as a reference. Simulations were carried out using the PMEMD engine with CUDA acceleration in AMBER22, while system topologies were prepared using antechamber and tleap.^35,36^ Each complex was solvated in an explicit TIP3P water box under periodic boundary conditions, with a minimum buffer of 10 Å from the protein surface. Energy minimization was conducted using 2500 steps of steepest descent, followed by additional minimization cycles with progressively reduced positional restraints and a final unrestrained minimization.^37,38^

The systems were heated to 300 K over 500 ps under an NVT ensemble, with restraints gradually relaxed to allow smooth thermal equilibration. Subsequent equilibration was performed in two stages, 1.5 ns under NPT conditions to stabilize pressure and temperature, followed by 3.5 ns under NVT conditions with stepwise removal of restraints. The final equilibration step was unrestrained, allowing free system relaxation at 300 K and 1 atm. Final production runs were then carried out for five complexes lasting up to 300 ns each. Temperature and pressure were maintained using Langevin dynamics and isotropic position scaling. Long-range electrostatics were treated using the Particle Mesh Ewald (PME) method, hydrogen-containing bonds were constrained using the SHAKE algorithm, and a 10 Å cutoff was applied for non-bonded interactions with a 2 fs time step.^39,40^ Trajectory analyses were performed using Chimera, VMD, and CPPTRAJ.^41–43^ Structural stability and flexibility were assessed through root mean square deviation (RMSD), root mean square fluctuation (RMSF), and radius of gyration (*R*_g_) analyses.^44^

### 2.10. Principal component analysis (PCA)

Principal Component Analysis (PCA) was used to simplify the complex data obtained from MD simulations by reducing its dimensionality, in order to reveal important details and patterns within the dataset. This analysis also provides details about the conformational alterations in proteins and derive significant insights from the complex motions evident in the MD trajectories. Trajectory alignment was performed as part of the standard preprocessing steps. The covariance matrix was diagonalized using the Essential Dynamics (ED) method *via* MDAnalysis tools^45^ to find eigenvectors, eigenvalues, and their projections, as:*C*_*ij*_ = (*r*_*i*_ − *r*_*i*_)(*r*_*j*_ − *r*_*j*_)

Diagonalizing this matrix (*C* = *VΛV*) yields the eigenvectors *V* (principal components, PC1 and PC2) which represent the directions of the largest variance in the protein's conformational space, and the eigenvalues *Λ* which represent the magnitude of those motions.

### 2.11. Free energy landscape (FEL)

The gmx sham module of the GROMACS software suite was used to generate the free-energy landscape based on the conformational space and molecular motions that were sampled during the simulations. The first two principal components were used to calculate Gibbs free energy profiles to illustrate the probability distribution of the molecular system during molecular dynamics simulations. As given in the following equation.^46^Δ*G* = −*K*_B_*T* ln *P*(PC_1_,PC_2_)

The probability distribution of the protein conformations with its two primary components is represented by *P*(PC_1_,PC_2_) in this equation, where *K*_B_ and *T* stand for the Boltzmann constant and absolute temperature, respectively. The free energy landscape provides a visual representation of the system's energy distribution, offering insight into molecular kinetics and thermodynamic stability by revealing distinct energy states and their associated conformational probabilities.

## 3. Results and discussion

### 3.1. CLM training and generation

The CLM was trained on approximately 2.3 million compounds obtained from ChEMBL, encoding 2 305 192 SMILES strings into the SELFIES representation, with only 34 sequences discarded due to conversion errors. The final vocabulary comprised of 301 unique tokens, and the resulting model contained approximately 1.8 million trainable parameters. Training was performed for 10 epochs using GPU hardware, enabling efficient mini-batch optimization. The model converged with a final token-level loss of 0.663 and a perplexity of 1.94, indicating that it effectively captured molecular syntax and sequential patterns within the training data. Model training was monitored across the 10 epochs, with training loss decreasing consistently from 0.883 to 0.663 and perplexity reducing from 2.42 to 1.94, demonstrating stable convergence without plateau or divergence (SI Fig. S1). Perplexity scores in language models is a measure of how confidently the model predicts the next token in a molecular sequence. In general, a lower value indicates higher predictive confidence. However, the interpretation of perplexity is highly dependent on the vocabulary size and structural constraints of the molecular representation used. SELFIES operate on a vocabulary that is approximately 1000 times smaller than natural language vocabularies, and unlike SMILES, the SELFIES grammar encodes chemical validity constraints directly into the representation so that any token sequence produces a valid molecule, reducing the uncertainty the model must resolve at each generation step.^47,48^ Perplexity values for SELFIES-based models are therefore inherently lower than those observed in SMILES-based or natural language models. During the generation phase, 2000 molecules were sampled autoregressively from the trained model, demonstrating its ability to produce diverse chemically valid structures. The SMILES representations of the generated molecules is provided in SI File 1.

### 3.2. Distributional similarity

To evaluate how well the generated molecules reproduced the statistical properties of real chemical space, the Fréchet ChemNet Distance (FCD) was computed between the generated set and the ChEMBL reference dataset. FCD compares neural network derived chemical feature distributions between generated molecules and a reference ChEMBL dataset. Lower FCD values indicate closer alignment with drug-like chemical space. The FCD of the ChEMBL drug database is widely used as the gold standard reference for drug-like molecules, and was also used as the standard benchmark for this study, with scores closer to 0 reflecting distributions most similar to known bioactive compounds. The proposed CLM achieved an FCD score of 0.93, indicating a high degree of alignment between the distributions of neural network-derived chemical features in the generated and reference molecules. This result is consistent with FCD values reported for established generative models in the literature, and suggests that the model effectively learned the latent rules of chemical validity and diversity, producing molecules that are not only valid and drug-like but also representative of real-world chemical diversity.^49,50^

### 3.3. Drug-likeness and chemical quality assessment

Post-generation analysis using RDKit indicated that all (100%) generated molecules were chemically valid. A substantial fraction of molecules (71.49%) satisfied Lipinski's Rule of Five, highlighting strong adherence to drug-likeness criteria. Only 2.91% of molecules contained PAINS substructures, suggesting minimal interference-prone motifs. The physicochemical properties of the generated molecules (mean ± SD) were noted as, molecular weight (MW) 392.01 ± 219.37 Da, log *P* 2.56 ± 2.51, topological polar surface area (TPSA) 91.42 ± 73.32 Å^2^, Quantitative Estimate of Drug-likeness (QED) 0.50 ± 0.25, and Synthetic Accessibility (SA) score 4.40 ± 1.49. These values indicate that the CLM produced molecules with diverse sizes, moderate lipophilicity, reasonable polarity, and manageable synthetic complexity, consistent with the broader chemical space of drug-like molecules in ChEMBL. On the whole, it was observed that the CLM efficiently learned chemical syntax and sequence patterns, generating a high fraction of chemically valid, structurally diverse molecules. The combination of strong Lipinski compliance, low PAINS incidence, and favorable QED and SA distributions suggest that the model produces chemically meaningful molecules suitable for further optimization. Additionally, to assess the novelty of the generated compounds a more rigorous literature-based approach was applied. The generated compounds were queried against the complete SciFinder database, a comprehensive repository of published chemical structures encompassing the broader scientific literature. SciFinder database queries confirmed that the majority of generated molecules (94%) represent novel chemical entities not previously reported in the literature, while a small number of generated structures (120 molecules) were found to match known compounds. This validates the model's ability to learn chemically meaningful antibacterial chemical space while predominantly generating genuinely novel scaffolds. The compounds short-listed for further studies, based on their novelty and satisfied drug like properties were considered. The RDKit profiles of all the generated molecules is provided as SI File 2.

### 3.4. ADME profiling

*In silico* ADME profiling was also performed using SwissADME on the set of CLM generated compounds, aimed to evaluate the pharmacokinetic plausibility of the selected molecules prior to structure-based prioritization. SwissADME predictions indicated predominantly moderate to high gastrointestinal absorption, supporting favorable oral bioavailability potential. Evaluation against multiple drug-likeness rules (Lipinski, Ghose, Veber, Egan, and Muegge) showed broad compliance, consistent with the initial RDKit-based filtering. Predicted solubility for most compounds ranged from soluble to moderately soluble. Overall, the SwissADME analysis confirms that the shortlisted CLM generated molecules possess pharmacokinetic properties suitable for downstream structure-based and experimental prioritization. The ADME profiles of the 96 molecules finally shortlisted for docking studies are provided as SI File 3.

### 3.5. Molecular interactions

Molecular docking was performed on the 96 shortlisted compounds while relebactam was used as a reference inhibitor to evaluate the binding profiles of the generated compounds against the KPC-2 active site using the co-crystal structure (PDB ID: 6QW9). Docking poses were systematically assessed based on predicted binding affinity, binding orientation, interaction profiles, and engagement with catalytically relevant residues within the active-site pocket. Particular emphasis was placed on interactions involving Ser70, which plays a central role as the nucleophilic residue responsible for β-lactam acylation and covalent inhibition by β-lactamase inhibitors. The docking results are provided as SI File 4.

**Fig. 2:**
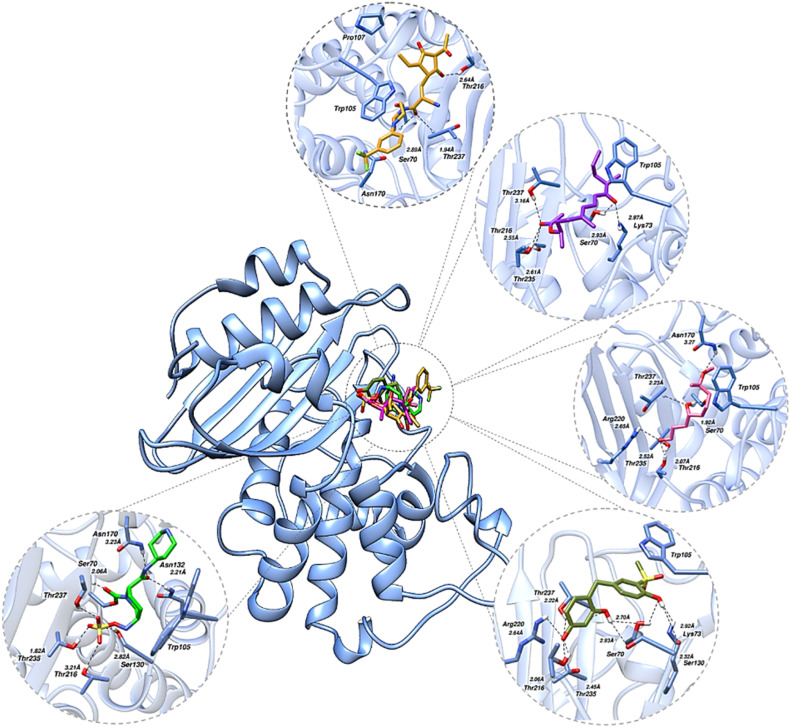
The binding profiles of the 4 shortlisted compounds, 46 (gold), 72 (purple), 75 (pink), and 88 (green), while the bottom panel shows the reference drug relebactam (green). KPC-2 is shown in cartoon representation with key active site residues rendered as sticks. Hydrogen-bonds are indicated by black dotted lines with distances in Å. Pictures were rendered utilizing UCSF Chimera.

Docking analysis identified four CLM-generated compounds, 46, 72, 75, and 88, exhibiting binding modes similar to that of the reference inhibitor relebactam within the KPC-2 active site. These candidates consistently adopted binding poses that positioned them near Ser70 and formed stabilizing interactions with multiple active-site residues, indicating a high likelihood of disrupting KPC-2 catalytic activity. As can be observed in Fig. 2, Compound 46 (gold) showed robust anchoring within the active site, forming a close interaction with the catalytic Ser70 (2.89 Å) and additional hydrogen-bonding contacts with Thr216 and Thr237, indicating effective occupation of the nucleophilic region critical for β-lactamase inhibition. Compound 72 (purple) adopted an extended binding orientation, establishing hydrogen bonds with Ser70 (2.93 Å) and Lys73 (2.97 Å), while also engaging Thr216 and Thr235, suggesting favorable stabilization within the catalytic cleft. Compound 75 (pink) exhibited particularly strong interactions, including a short hydrogen bond with Ser70 (1.96 Å) and additional contacts with Thr216, Thr235, and Arg220, reflecting a well-coordinated interaction network across the active site. Compound 88 (green) similarly engaged Ser70 (2.93 Å) and Lys73, while maintaining interactions with Thr216 and Thr235, consistent with effective positioning near the catalytic serine. In comparison, relebactam formed canonical interactions with Ser70, Thr216, Thr235, and Asn170, serving as a benchmark for productive KPC-2 inhibition. Remarkably, all four shortlisted compounds recapitulated these critical interaction patterns. These findings suggest compounds 46, 72, 75, and 88 as *in silico* prioritized KPC-2 inhibitor candidates with binding modes closely resembling that of relebactam. These compounds were then considered for further computational analyses.

All four lead compounds were found engaging the catalytic Ser70 of KPC-2, confirming it as the primary pharmacophoric anchor of the series. Compound 46 employs its dihydroxylated cyclohexenone core as a H-bond donor for Ser70, Thr216, and Thr237, while its trifluoromethylphenyl group provides a lipophilic anchor at Pro107. Compound 72 engages five residues through ketone and ester carbonyls as H-bond acceptors, while hydrophobic contacts at Trp105 and Thr216 were observed. Compound 75, despite its structurally simple aliphatic diol scaffold, demonstrates the broadest polar interaction network, forming the shortest H-bond with Ser70 (1.92 Å) and additionally engaging Asn170 and Arg220, interactions driven by optimally positioned hydroxyl groups rather than molecular complexity. Compound 88 was found pharmacophorically the most complete inhibitor of the series, uniquely engaging the catalytic residues (Ser70, Lys73, Ser130) alongside Arg220 and Trp105, the methylsulfonyl group serves as a critical H-bond acceptor complementary to Arg220, while three phenolic hydroxyl groups collectively occupy the catalytic machinery. The Trp105-facing hydrophobic subpocket emerges as a conserved secondary pharmacophoric feature across compound 72, compound 75, and compound 88, identifying it as a key target for scaffold optimization. Regarding synthetic feasibility, compound 75 represents the most accessible candidate requiring minimal synthetic steps from commercial chiral precursors, compound 88 is accessible *via* acid-catalyzed bisphenol condensation and sulfonylation, while compound 46 and compound 72 involve moderate complexity through amide coupling and stereocontrolled polyene synthesis respectively, all representing practically achievable routes. A detailed retrosynthetic analysis will be considered in future experimental work.

### 3.6. Toxicity profiling

*In silico* toxicity profiling of the four *in silico* prioritized compounds was performed using ProTox-III. Compound 46 and compound 72 were assigned toxicity class IV, with predicted LD_50_ values of 1780 mg kg^−1^ and 2000 mg kg^−1^ respectively, indicating they are harmful if swallowed. Compound 88 and compound 75 were assigned toxicity class V, with predicted LD_50_ values of 3160 mg kg^−1^ and 5000 mg kg^−1^ respectively, indicating they may be harmful if swallowed. Of these, compound 75 demonstrated the most favorable predicted oral toxicity profile with the highest LD_50_ of 5000 mg kg^−1^. Notably, class IV and V classifications are consistent with the oral toxicity profiles of several clinically approved antibacterial agents, especially, in the context of KPC-2 inhibitors such as relebactam which are administered intravenously in clinical practice. These findings collectively suggest that these *in silico* prioritized lead compounds do not exhibit alarming predicted toxicity, however, comprehensive experimental toxicity evaluation remains an essential prerequisite for any future preclinical development.

### 3.7. Molecular dynamics stability and flexibility analysis

The structural stability and dynamic behavior of the KPC-2 complexes bound to the reference inhibitor relebactam and the top-ranked generated compounds (46, 72, 75, and 88) were evaluated using 300 ns all-atom MD simulations. System stability was assessed through protein backbone RMSD, ligand RMSD, residue-wise RMSF, and radius of gyration (*R*_g_) analyses.

**Fig. 3:**
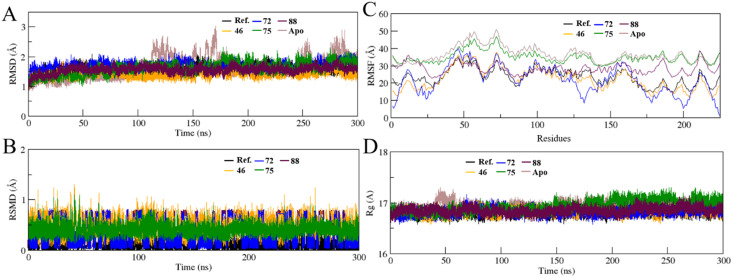
Molecular dynamics stability analysis of KPC-2 complexes with relebactam (black) and compounds 46 (orange), 72 (blue), 75 (green), and 88 (maroon). Shown are (A) protein backbone RMSD, (B) ligand RMSD, (C) residue-wise RMSF, and (D) radius of gyration (*R*_g_) over 300 ns simulations, demonstrating overall structural stability and sustained ligand binding across all complexes.

Protein backbone RMSD (Fig. 3A) shows that all systems rapidly converged within the initial 20–30 ns and remained stable throughout the simulation period. The backbone RMSD values were consistently maintained within 1.3–2.1 Å for all complexes, indicating no major structural deviations from the starting conformations. The relebactam–KPC-2 complex exhibited RMSD values centered around 1.5–1.7 Å. Similar stability profiles were observed for compounds 46 and 72, while compound 75 showed slightly higher fluctuations toward the later stages (∼2.0 Å), though still within an acceptable range for stable protein–ligand complexes. Compound 88 closely followed the reference system, indicating strong conformational stability. The apo protein exhibited high variability throughout the simulation, with fluctuations reaching up to 3 Å after 100 ns, reflecting the structural instability of the unoccupied binding pocket in the absence of a ligand.

Ligand RMSD (Fig. 3B) further confirmed binding stability within the active site. Relebactam showed ligand RMSD values largely below 0.3 Å after equilibration, reflecting a tightly bound and stable pose. Among the generated candidates, compounds 72 and 88 exhibited similarly low RMSD values (0.4 Å), while compounds 46 and 75 showed slightly higher fluctuations (0.6–0.9 Å). All ligands remained stably anchored within the KPC-2 binding pocket during the entire duration of the simulation.

Residue-wise RMSF analysis (Fig. 3C) revealed that fluctuations were largely confined to loop regions, while the core secondary structure elements remained rigid. Most active-site residues exhibited RMSF values below 25 Å. Importantly, regions surrounding catalytically critical residues such as Ser70, Lys73, Thr216, Thr235, and Trp105 showed limited flexibility across all systems. Compounds 72 and 88 induced lower fluctuations in the active-site region when compared with compound 75, indicating better local stabilization similar to the reference relebactam complex. The apo protein demonstrated high residue fluctuations across most regions compared to the ligand–bound complexes, confirming that ligand binding effectively reduces the conformational flexibility of KPC-2.

Radius of gyration (*R*_g_) profiles (Fig. 3D) demonstrated that all complexes retained a compact global fold throughout the simulations. The *R*_g_ values remained stable within 16.6–17.2 Å, with minor fluctuations over time. The reference system showed an average *R*_g_ of 16.8 Å, closely matched by compounds 46, 72, and 88. Compound 75 exhibited a slightly higher *R*_g_ toward the latter half of the simulation (17.1–17.3 Å), suggesting a marginal increase in overall flexibility without loss of structural integrity. The apo protein displayed a slight initial elevation in *R*_g_ during the first 50 ns of simulation before stabilizing, indicative of transient structural expansion in the unoccupied state, while all ligand–bound complexes maintained consistently compact and stable *R*_g_ values of approximately 16.8–17.0 Å throughout the entire simulation period.

These results indicate that the CLM-generated compounds, particularly 46, 72, and 88, exhibit dynamic stability, conformational behavior, and protein compactness in a similar manner to the clinically approved inhibitor relebactam. The consistency across RMSD, RMSF, and *R*_g_ metrics supports the formation of stable protein–ligand complexes and reinforces the suitability of these candidates for further optimization and experimental validation.

### 3.8. Conformational motions and thermodynamic landscape

**Fig. 4:**
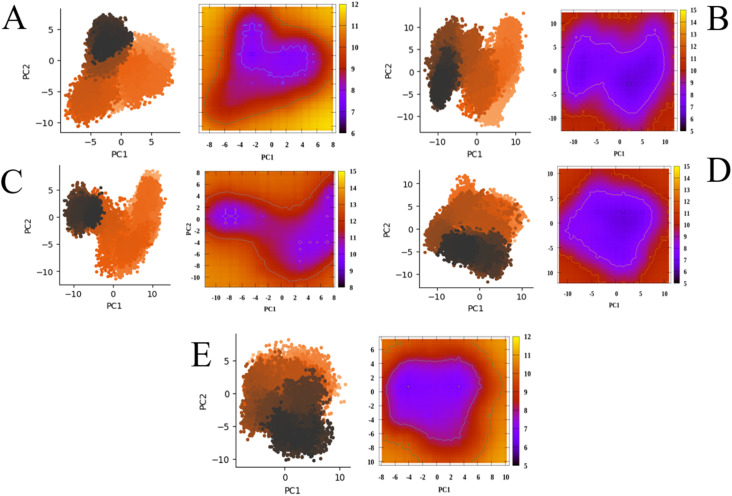
The PCA and FEL profiles of the reference compound (A) relebactam and compounds (B) 46, (C) 72, (D) 75, and (E) 88 in complex with KPC-2.

The principal component analysis (PCA) plot provides a detailed visualization of the significant conformational of KPC-2 in complex with the reference inhibitor relebactam and the shortlisted compounds (46, 72, 75, and 88). The first two principal components (PC1 and PC2), which captured the majority of the conformational variance, were used to construct two-dimensional conformational projections and corresponding free energy landscapes (FEL). A side by side representation of the PCA and FEL profiles for all KPC-2 protein–ligand systems are illustrated in Fig. 4A–E, highlighting their conformational and thermodynamic stability.

The KPC-2–relebactam complex (Fig. 4A) display a compact PCA distribution, largely confined within PC1 values of approximately −4 to +4 and PC2 values between −3 and +3, while the corresponding FEL reveals a well-defined global minimum with free energy values around 6–8 kcal mol^−1^, indicating a dominant and stable conformational state throughout the simulation. Complexes of KPC-2 with compounds 46, 72, and 75 (Fig. 4B–D) show a slightly broader PCA sampling compared with the reference, with PC1 ranges extending up to −8 to +8 and PC2 up to −7 to +7. Despite this increased flexibility, the conformational space remains organized into distinct clusters rather than diffused distributions. The corresponding FELs exhibit clearly defined low-energy basins, with minimum free energy regions ranging from approximately 7 to 10 kcal mol^−1^, suggesting the presence of stable, energetically favorable binding states. Compound 88 (Fig. 4E) showed a more dispersed PCA, spanning a wider region of conformational space. The corresponding FEL appear flatter, with broader low-energy regions and less defined minima, indicative of increased conformational heterogeneity and reduced stabilization relative to the other candidates. These PCA and FEL analyses demonstrate that compounds 46, 72, and 75 show conformational and binding free energy profiles similar to the reference inhibitor, maintaining confined low-energy conformational states during simulation, while compound 88 exhibits greater conformational freedom.

## 4. Conclusion

This study presents a comprehensive computational strategy for the discovery of novel inhibitors against KPC-2 β-lactamase, a key driver of carbapenem resistance in *Klebsiella pneumoniae* and other ESKAPE pathogens. By integrating a SELFIES-based recurrent CLM with multiple filtering criteria, structure-based docking, and molecular dynamics simulations, an efficient pipeline for exploring biologically relevant chemical space is proposed. This generative model exhibited stable training behavior and high syntactic fidelity, producing molecules that closely align with known drug-like chemical space, as reflected by an FCD score of 0.93, complete RDKit validity, and favorable drug-likeness characteristics. The compound shortlisting was based on a multi-parameter assessment including Lipinski's rule, PAINS substructure screening, Quantitative Estimate of Drug-likeness (QED), Synthetic Accessibility (SA) score, and topological polar surface area (TPSA), and *in silico* ADME profiling. Additional filters including the Pfizer 3/75 rule, GSK 4/400 rule, and Golden Triangle rule, will also be included in future work to further refine candidate selection and better flag compounds with unfavorable toxicity and pharmacokinetic profiles.

Subsequent structure-based screening and dynamic analysis identified compounds 46, 72, 75 and 88 as particularly promising candidates based on *in silico* prioritization, exhibiting stable binding modes, favorable interactions with key catalytic residues in the KPC-2 active site, and dynamic behavior similar to that of the clinically approved inhibitor relebactam in terms of interactions. While relebactam was selected as the reference compound based on its clinical relevance as the most efficacious approved inhibitor against KPC-2-producing *Klebsiella pneumoniae*, the use of a single reference compound represents a methodological limitation of this study. Future work will incorporate additional β-lactamase inhibitors, such as avibactam and vaborbactam, as reference standards to enable more comprehensive benchmarking of the computationally predicted candidates. These findings underscore the ability of generative CLMs to produce chemically valid, synthetically feasible, and biologically relevant molecules when coupled with rigorous post-generation evaluation. While several CLMs have already been established using Transformer architectures, these models are often large, computationally intensive, and require substantial GPU resources and extensive datasets. In contrast, our LSTM-based CLM is lightweight, transparent, and fully reproducible, relying on SELFIES to ensure chemical validity. This simplicity allows efficient large-scale molecular generation on modest hardware while maintaining competitive distribution-level performance, motivating the development of a new CLM tailored for robust and scalable drug-like molecule generation. While the strong generative performance metrics support the adequacy of the single-layer design for SELFIES-based antibacterial molecule generation, future work will explore the effect of architectural depth on generative diversity and chemical space coverage.

This study thereby establishes a comprehensive computational framework for the preliminary discovery of KPC-2 inhibitors and highlights the broader potential of CLMs to accelerate antibiotic discovery. The proposed approach is readily generalizable to other resistance determinants, offering a versatile strategy to address the growing global challenge of antimicrobial resistance. However experimental validation remains an essential validation step. Future work, focused on the synthesis of *in silico* prioritized candidates and their biological evaluation against KPC producing *Klebsiella pneumoniae* strains is also planned to further validate and refine our computational predictions. These studies will collectively establish the translational potential of the CLM driven framework proposed in this work.

## Conflicts of interest

The authors declare no conflicts of interest.

## Supplementary Material

RA-016-D6RA02379G-s001

RA-016-D6RA02379G-s002

RA-016-D6RA02379G-s003

RA-016-D6RA02379G-s004

RA-016-D6RA02379G-s005

## Data Availability

All data is available within the article and supplementary information (SI). Supplementary information: SuppFigS1_LossCurves, SuppFile1_GeneratedSmiles, SuppFile2_RdKitReport, SuppFile3_SwissADME and SuppFile4_DockingResults. See DOI: https://doi.org/10.1039/d6ra02379g.
