# Supplementary material for: Data-driven design and screening of novel Klebsiella pneumoniae carbapenemase-2 β-lactamase inhibitors using a generative CLM
Source: RSC Adv. 2026 May 26;16(31):28845–56. doi: 10.1039/d6ra02379g (PMC13213580; doi:10.1039/d6ra02379g)
Supplement: RA-016-D6RA02379G-s001 [file RA-016-D6RA02379G-s001.pdf]

Supplementary Information (SI) for RSC Advances.
This journal is © The Royal Society of Chemistry 2026

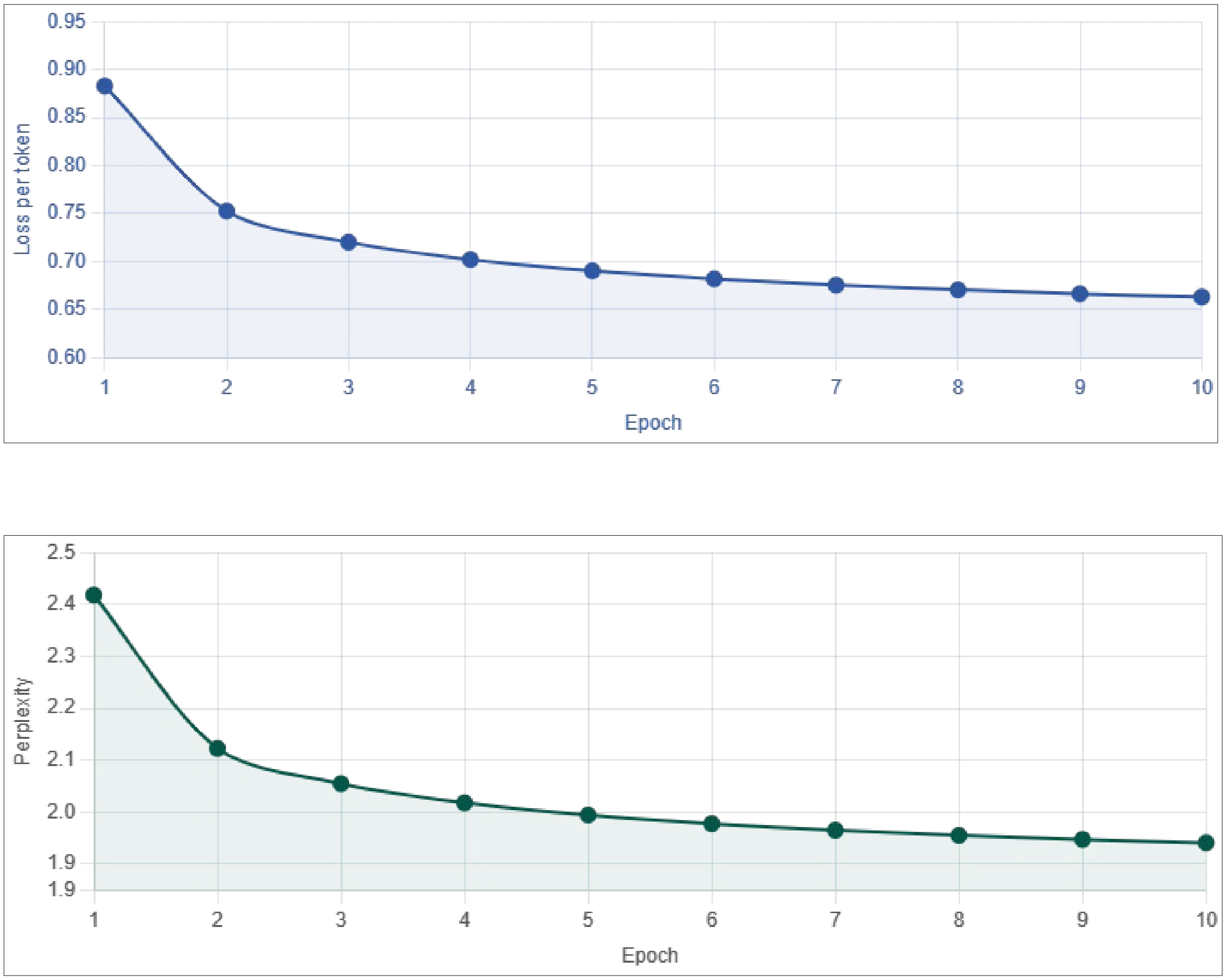

Figure S1: Training loss and perplexity curves across all 10 epochs, demonstrating smooth and consistent convergence without plateau or divergence. The gradual decrease in training loss from 0.883 at epoch 1 to 0.663 at epoch 10, alongside perplexity reduction from 2.42 to 1.94, shows stable model learning.
